# Effects of Ion Characteristics on the Leaching of Weathered Crust Elution-Deposited Rare Earth Ore

**DOI:** 10.3389/fchem.2020.605968

**Published:** 2020-12-15

**Authors:** Zhenyue Zhang, Ru'an Chi, Zhuo Chen, Wendou Chen

**Affiliations:** ^1^School of Xingfa Mining Engineering, Wuhan Institute of Technology, Wuhan, China; ^2^Key Laboratory for Green Chemical Process of Ministry of Education, Wuhan Institute of Technology, Wuhan, China

**Keywords:** weathered crust elution-deposited rare earth ores, cation, anion, leaching efficiency, zeta potential, swelling ratio

## Abstract

To reveal the ion-exchange mechanism in the leaching process of weathered crust elution-deposited rare earth ores with different leaching agents, the effects of a variety of cations and anions at different concentrations on the leaching process were investigated, including Al^3+^, Fe^3+^, Ca^2+^, Mg^2+^, Na^+^, K^+^, ${\text{NH}}_{4}^{+}$ and Cl^−^, ${\text{NO}}_{3}^{-}$, and ${\text{SO}}_{4}^{2-}$. Meanwhile, the relationships between different concentrations of cations and anions and leaching efficiency were investigated, as was the relationship between different concentrations of cations and anions and zeta potential. The effect of different ions on the swelling of clay minerals during leaching process was also investigated. The results shown that ${\text{NH}}_{4}^{+}$ was the most affected electrolyte cation in terms of rare earth leaching efficiency during the leaching process of weathered crust elution-deposited rare earth ore among three different cationic valence states, and the leaching efficiency was 86.93% at the optimal leaching concentration. The influence of the three anions on the leaching efficiency of rare earth was ${\text{NO}}_{3}^{-}>{\text{Cl}}^{-}>{\text{SO}}_{4}^{2-}$, and the leaching efficiency of rare earth were 83.21, 81.52, and 80.12% at the optimal leaching concentration, respectively. The ${\text{NH}}_{4}^{+}$ had the greatest effect on the zeta potential of weathered crust elution-deposited rare earth ore, and the zeta potential was −18.1 mV at the optimal leaching concentration. Additionally, the order of the effect of three anions on zeta potential was ${{\text{SO}}_{4}^{2-}>\text{NO}}_{3}^{-}>{\text{Cl}}^{-}$. Combined with the effect on the rare earth leaching process, anions and cations were considered separately, and ${\text{NH}}_{4}^{+}$ and Cl^−^ were selected; the relationship between the rare earth leaching efficiency and zeta potential conforms to the follow equations: ${\text{NH}}_{4}^{+}$:Y = −0.48X^2^ – 13.51X – 1.58, *R*^2^ = 0.98133 and Cl^−^:Y= −1.22X^2^ – 17.64X + 23.29, *R*^2^ = 0.99010. It was also found in the swelling experiment of the weathered crust elution-deposited rare earth ore that the swelling ratio of clay minerals was the lowest when the cation and anion were ${\text{NH}}_{4}^{+}$ and Cl^−^ and the swelling ratios were 1.874 and 2.015%, respectively.

## Introduction

Weathered crust elution-deposited rare earth ore is of extremely high economic and strategic value, it is a special rare earth resource in the world (Simandl, 2014; Zhang B. et al., 2018; Zou et al., 2020). As a unique resource, it contains almost rare earth elements, including the light rare earth and middle and heavy rare earth, being especially rich in middle and heavy rare earth (Xiao et al., 2015a; Chi and Liu, 2019). The rare earth elements mainly existed in the ionic or hydrated ionic state on the surface of the clay minerals (Huang et al., 2005). Due to this special property, the essential process during leaching is an ion exchange reaction between the surface of clay minerals of absorbed rare earth ions and cations in lixiviant. Rare earth ions were obtained by way of an ion exchange reaction, and this method has been applied in industry. In addition, the *in-situ* leaching process, with ammonium sulfate as the main leaching agent, was gradually formed (Chi and Wang, 1993; Chi et al., 2012). However, the leaching process causes the swelling of clay minerals, which has a certain influence on the safety of mine production and the low efficiency of the leaching agents, resulting in high consumption of electrolyte solution in actual industrial production.

In order to improve the efficiency of the leaching process and mine safety, a large number of studies have been conducted on the rare earth leaching process of weathered crust elution-deposited rare earth ores under various leaching systems (Xiao et al., 2015b). Zhang et al. (2013) and He et al. (2016) found that the order of the leaching efficiency of rare earth by inorganic ammonium salt was (_NH_4_)2_SO_4_ < NH_4_*Cl* < NH_4_NO_3_, which was related to the complexation capacity between rare earth ions and anions. Chen et al. (2018a) and Chen Z. et al. (2020) found that the order of mass transfer efficiency of rare earth under three magnesium salts was Mg(_NO_3_)2_ > Mg(Cl)_2_ > MgSO_4_ in the study of mass transfer process of weathered crust elution-deposited rare earth ores by magnesium salts. The inhibiting swelling effect was highest with magnesium nitrate as the leaching agent, and the mass transfer efficiency of rare earth was related to the type of anion. Li et al. (2019) proposed that the cationic activity of the leaching agent was an important factor affecting the leaching quality of rare earth, and the molar concentration of the leaching agent affected the leaching efficiency to a certain extent. The effects of different electrolyte solutions on leaching were considered. Zhang Z. Y. et al. (2018) studied ammonium salt leaching weathered crust elution-deposited rare earth ores and found in the study that the zeta potential of rare earth ore decreased with the increase of leaching solution pH, and the zeta potential increased with the increase of ammonium concentration. Besides, zeta potentials of rare earth ores with AlCl_3_, NH_4_Cl, KCl, and MgCl_2_ solution were negative. Wang et al. (2018) studied the effect of ion interaction on rare earth leaching, and they mentioned that the leaching capacity of leaching agent on rare earth at low concentration was consistent with the adsorption capacity of cation; ${\text{SO}}_{4}^{2-}$ showed stronger coordination and leaching aid than Cl^−^ with rare earth ions. On the other hand, when the leaching agent concentration increased, the electrostatic interaction between ions will inhibit the leachate of rare earth, especially for the higher cations. Therefore, it is necessary to consider the anionic and cationic properties in the leaching process.

There were many studies on the weathered crust elution-deposited rare earth ore with single cation under different experimental conditions. However, the leaching process of weathered crust elution-deposited rare earth ores with different anions and cations was rarely studied systematically. Seven common cations and three anions were therefore selected to study the effects of ion property on the leaching efficiency of rare earth and zeta potential of rare earth ore in this paper, including Al^3+^, Fe^3+^, Ca^2+^, Mg^2+^, Na^+^, K^+^, and $\;{\text{NH}}_{4}^{+}$ as well as Cl^−^, ${\text{NO}}_{3}^{-}$, and ${\text{SO}}_{4}^{2-}$. Meanwhile, the effect of ion property on swelling ratio of rare ore was also investigated. The effects of different cation and anion concentration on leaching process of weathered crust elution-deposited rare earth ores and zeta potential were studied. The relationship between the zeta potential of clay mineral and rare earth leaching efficiency were discussed. The effects of various ions on the swelling properties of clay minerals were also discussed. It would also enrich the study of the leaching process of weathered crust elution-deposited rare earth ores with electrolyte solution and provide theoretical guidance for efficient recovery of rare earth.

## Materials and Methods

### Materials

#### Chemical Composition

The weathered crust elution-deposited rare earth ore samples were collected from Fujian Province, China. The main chemical composition of the rare earth ore was analyzed by X-ray fluorescence (Axios advanced, Panalytical B.V.), and the results are shown in Table 1.

**Table 1:** Main chemical composition of the RE ores (mass fraction, %).

**Component**	**REO**	**SiO_**2**_**	**Al_**2**_O_**3**_**	**Fe_**2**_O_**3**_**	**K_**2**_O**	**Na_**2**_O**	**MgO**	**CaO**
Content	0.129	51.236	37.649	4.033	2.318	0.062	0.536	0.382
Component	TiO_2_	MnO	ZrO_2_	Rb_2_O	ZnO	CuO	SrO	Loss
Content	0.398	0.052	0.011	0.013	0.005	0.006	0.01	3.207

It can be seen from Table 1 that SiO_2_ and Al_2_O_3_ were the main chemical components of the sample, accounting for 51.236 and 37.649% of the total mass, respectively, and the REO content was 0.129% in the ore samples.

#### Rare Earth Partitioning

**Figure 1:**
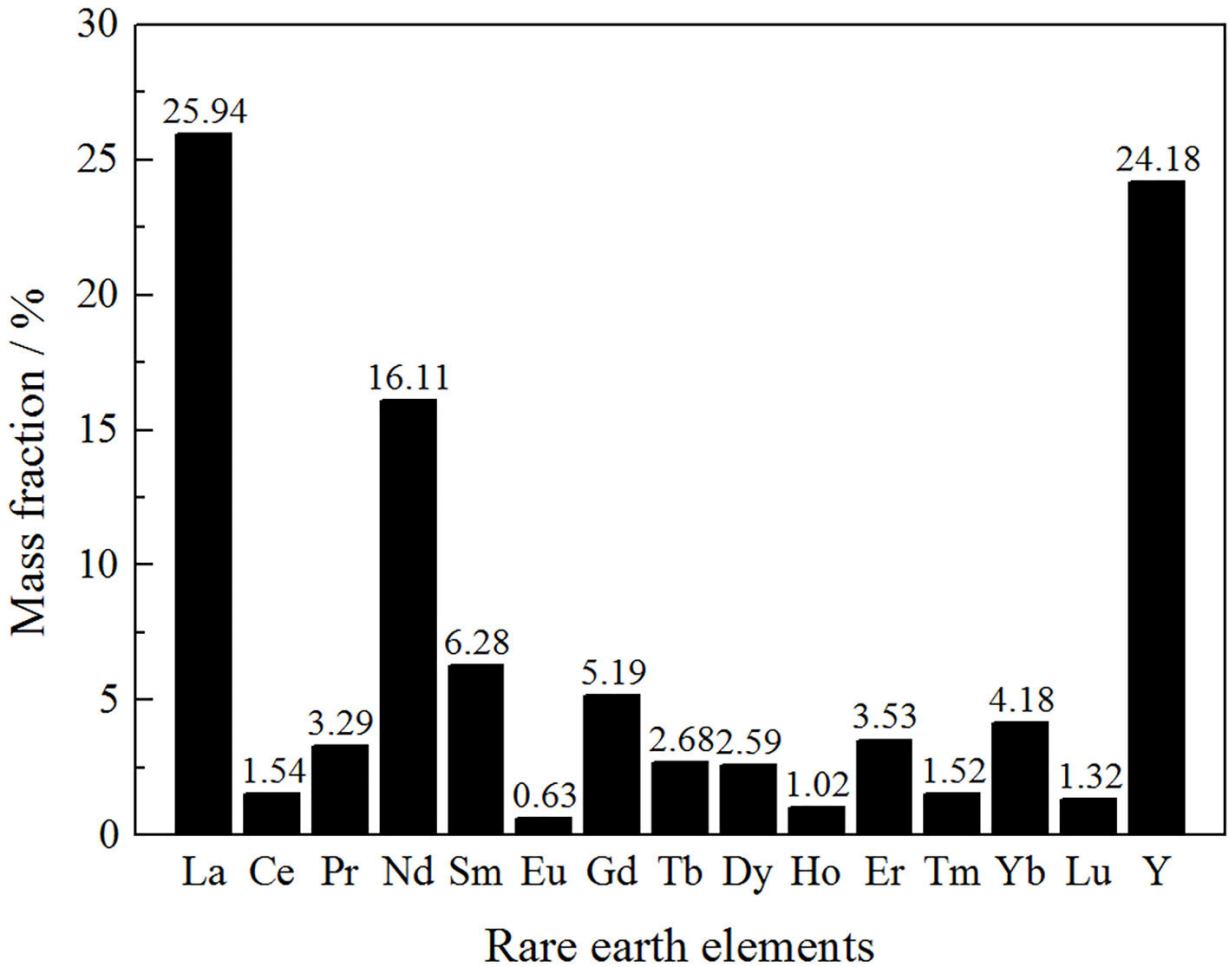
Rare earth elements of the ore sample.

Rare earth partitioning is an important index to evaluate the industrial value of the rare earth ore (Zhang et al., 2016a,b). The rare earth elements partitioning of the rare earth ore was analyzed by ICP-MS (Inductively Coupled Plasma Mass Spectrometry), as shown in Figure 1.

As can be seen from Figure 1, the content of light rare earths was 46.88% in the total rare earth content. The remaining medium and heavy rare earths accounted for 53.12%, which indicated that this rare earth mine has a great industrial utilization value.

### Apparatus and Experimental Procedure

#### Leaching Mechanism of Rare Earth Ore

The ion exchange reaction occurs between the rare earth ions and different valence cations can be represented by the following equations (Chi et al., 2012):

(1) $$\begin{array}{l} {\left[{\text{Al}}_{2}{\text{(Si}}_{2}{\text{O}}_{5}{\text{)(OH)}}_{4}\right]}_{\text{m}}{\text{nRE}}^{\text{3 + }}{\text{(s) + 3nA}}^{\text{ + }}\text{(a)}\\ \;={\left[A{\text{l}}_{2}{\text{(Si}}_{2}{\text{O}}_{5}{\text{)(OH)}}_{4}\right]}_{\text{m}}{\text{3nA}}^{\text{ + }}{\text{(s) + nRE}}^{\text{3 + }}\text{(a) } \end{array}$$

(2) $$\begin{array}{l} {\left[{\text{Al}}_{2}{\text{(Si}}_{2}{\text{O}}_{5}{\text{)(OH)}}_{4}\right]}_{\text{m}}{\text{2nRE}}^{\text{3 + }}{\text{(s) + 3nB}}^{2+}\text{(a)}\\ \;={\left[A{\text{l}}_{2}{\text{(Si}}_{2}{\text{O}}_{5}{\text{)(OH)}}_{4}\right]}_{\text{m}}{\text{3nB}}^{2+}{\text{(s) + 2nRE}}^{\text{3 + }}\text{(a) } \end{array}$$

(3) $$\begin{array}{l} {\left[{\text{Al}}_{2}{\text{(Si}}_{2}{\text{O}}_{5}{\text{)(OH)}}_{4}\right]}_{\text{m}}{\text{nRE}}^{\text{3 + }}{\text{(s) + nC}}^{3+}\text{(a)}\\ \;={\left[A{\text{l}}_{2}{\text{(Si}}_{2}{\text{O}}_{5}{\text{)(OH)}}_{4}\right]}_{\text{m}}{\text{nC}}^{3+}{\text{(s) + nRE}}^{\text{3 + }}\text{(a)} \end{array}$$

where s represents the solid phase and a represents the liquid phase.

As shown in the equation, minerals [A_l_2_(Si_2_O_5_)(OH)_4_ ] m_ can be described as resin, which adsorbed *RE*^3+^. When leaching agent flow through the resin, different valence cations migrate to the mineral surface and then take ion-exchange reaction with *RE*^3 +^.

#### Experimental Procedure

**Figure 2:**
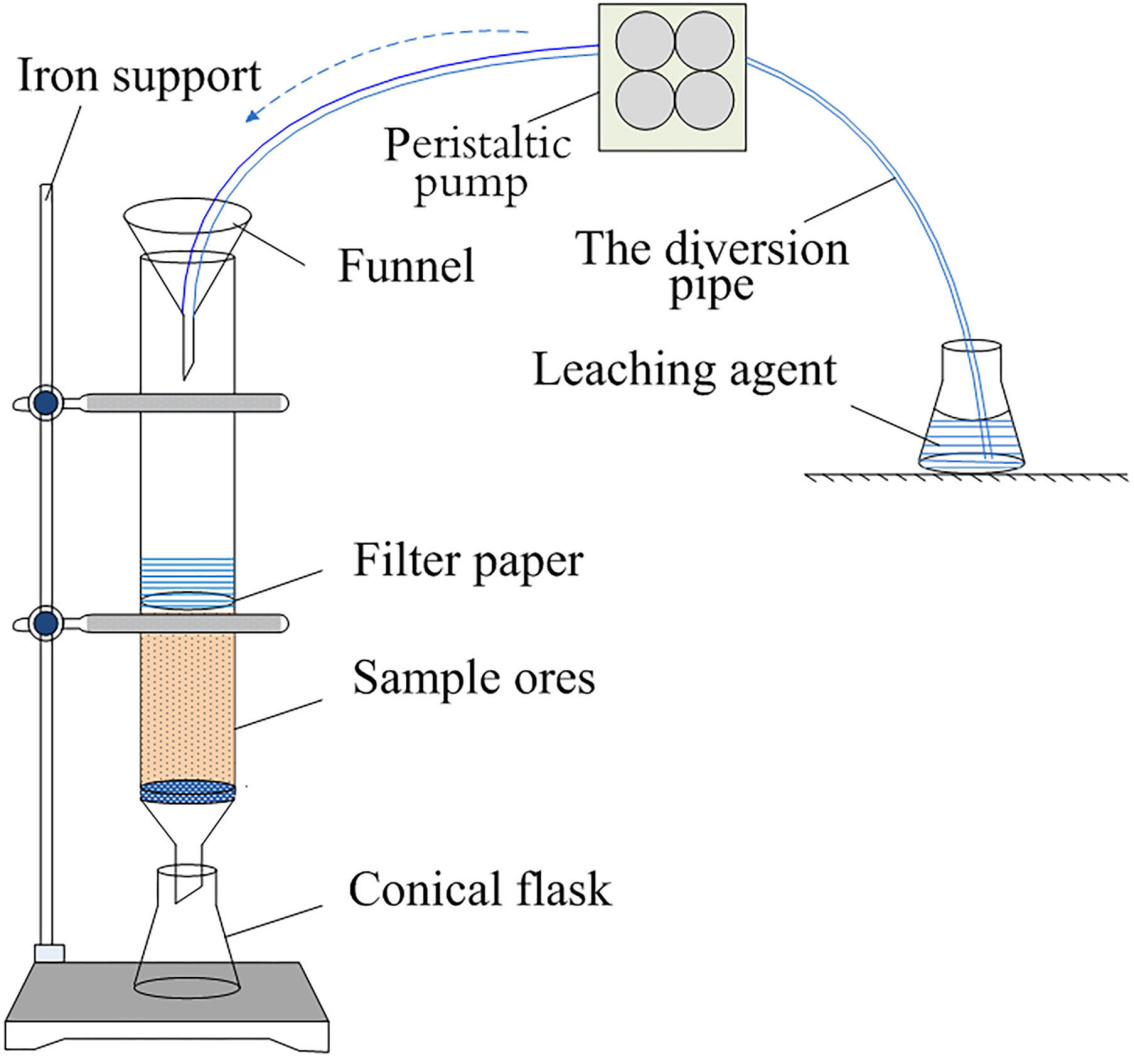
Self-made leaching device.

The column leaching method was adopted to simulate the *in-situ* leaching technology in the paper. The dried ore sample was weighed to 250 g by the quartering method and packed into the column tightly. The leaching agent was added at the ratio of 2:1 for liquid/solid. The leaching agent was added into the glass column under the control of precision pump at a speed of 0.5 mL/min (Chen et al., 2018b). The self-made leaching device was shown in Figure 2. The lixivium was collected into centrifuge tube from the bottom of the glass column, the rare earth content was analyzed by ICP-MS, and the rare earth leaching efficiency was calculated according to the follow formula (Yang et al., 2018):

(4) $$L(\%)=\frac{\left(C\times V\right)}{W\times C_{S}}\times 100$$

where L is the rare earth leaching efficiency/ %, C is the concentration of rare earth in leaching lixivium/ g/L^−1^, V is the volume of leaching lixivium/ L, W is the weight of rare earth ore sample/ g, and Cs is the content of rare earth in ionic phase of the ore samples/ %

**Figure 3:**
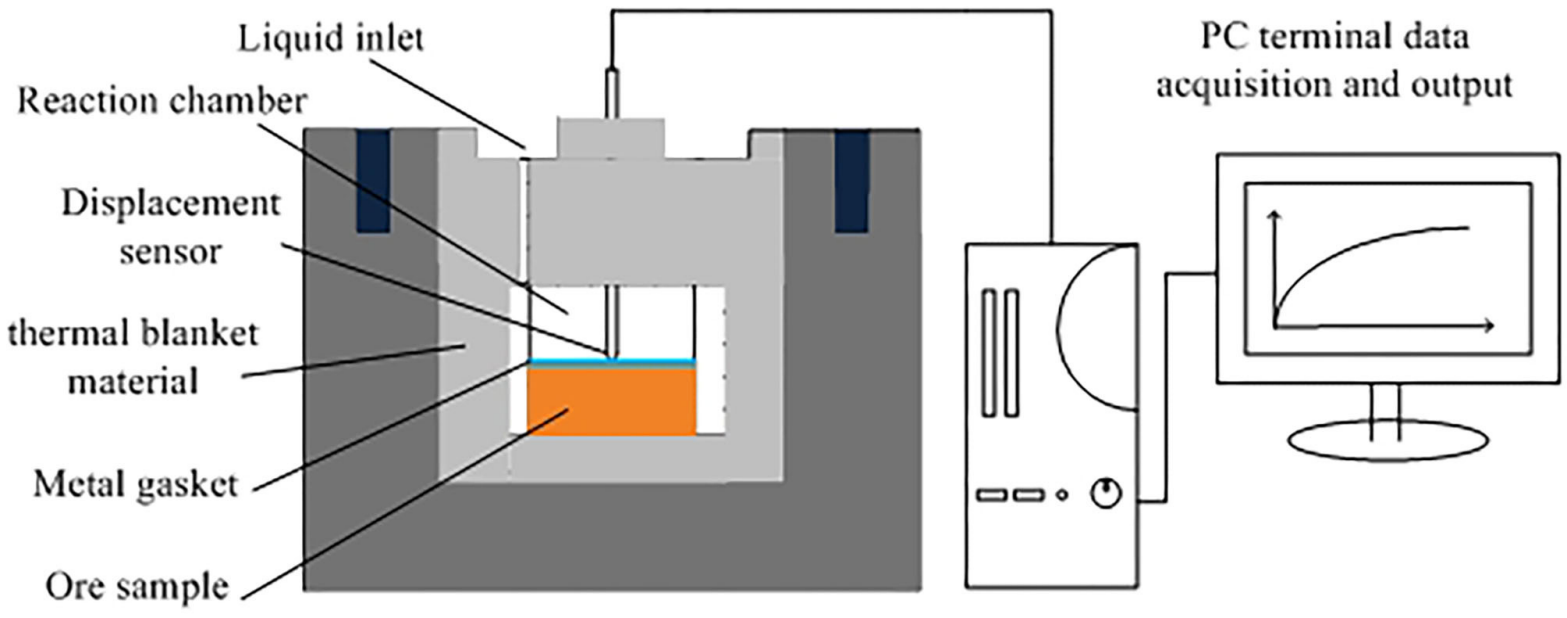
PCY intelligent dilatometer.

The swelling of clay mineral was conducted by PCY intelligent dilatometer. A total of 2 g of ore sample was weighed and pressed for 5 min with a tablet press under a pressure of 8 MPa. The initial length of the sample was recorded, and it was loaded it into a PCY intelligent clay dilatometer to determine its swelling ratio. The PCY intelligent dilatometer is shown in Figure 3. The swelling ratio of the ore sample was measured by the following formula (Zhang et al., 2013; Chen W. D. et al., 2020):

(5) $$\begin{array}{r} \delta =\frac{\Delta H}{H_{0}}\times 100 \end{array}$$

where *H* is the variation of height/ mm, and *H*_0_ is the initial height of the ore sample/ mm

The zeta potential was detected by Zetasizer Nano (Malvern Instruments Corporate, UK).

All chemicals used in the experiments were of analytical grade. All the solutions were prepared with deionized water.

## Results

### Relationship Between Rare Earth Leaching Efficiency and Zeta Potential of Rare Earth Ore

**Figure 4:**
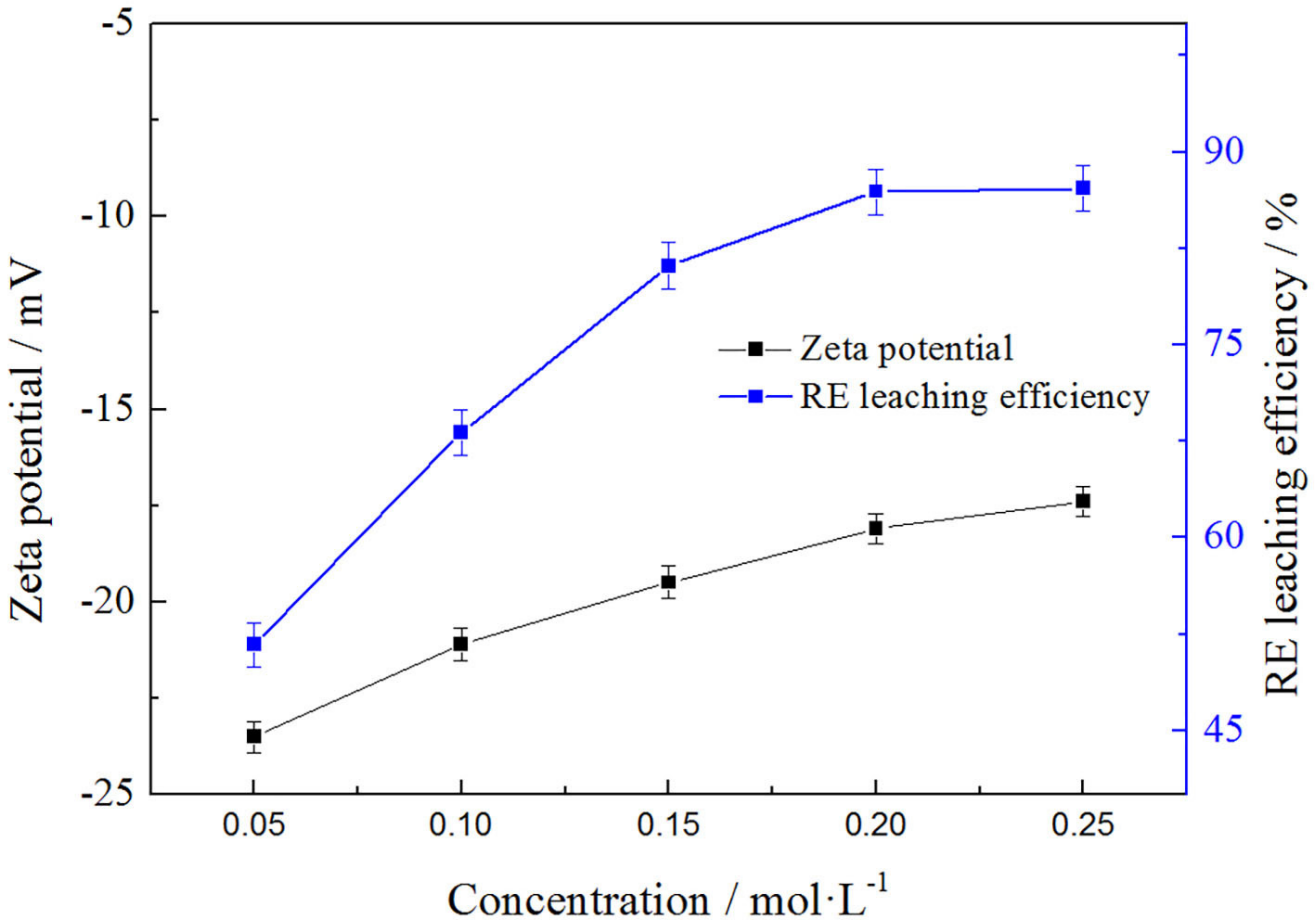
Relationship between zeta potential and rare earth efficiency.

In order to analyze the relationship between the rare earth leaching efficiencies and zeta potentials of clay minerals with leaching agent, the effects of *NH*_4_*Cl* concentrations on the rare earth leaching were investigated. The result is shown in the Figure 4.

It can be seen from the Figure 4 that the rare earth leaching efficiencies and zeta potentials of rare earth both increased with the increase of leaching agent concentration, and the rate of increase was both fast and then slow when the ammonium concentration exceeded 0.2 mol/L and finally tended to keep balance to some extent. The rare earth leaching efficiency was basically balanced when the solvent concentration was 0.2 mol/L and the leaching efficiency was 86.93%. At that time, the zeta potential on the clay mineral surface was −18.1 mV, which was also close to the equilibrium value. It was obvious that the rare earth leaching efficiencies and zeta potentials displayed similar trends in the leaching process. To better understand this phenomenon, the electronic double-layer theory was used to analyze the results.

**Figure 5:**
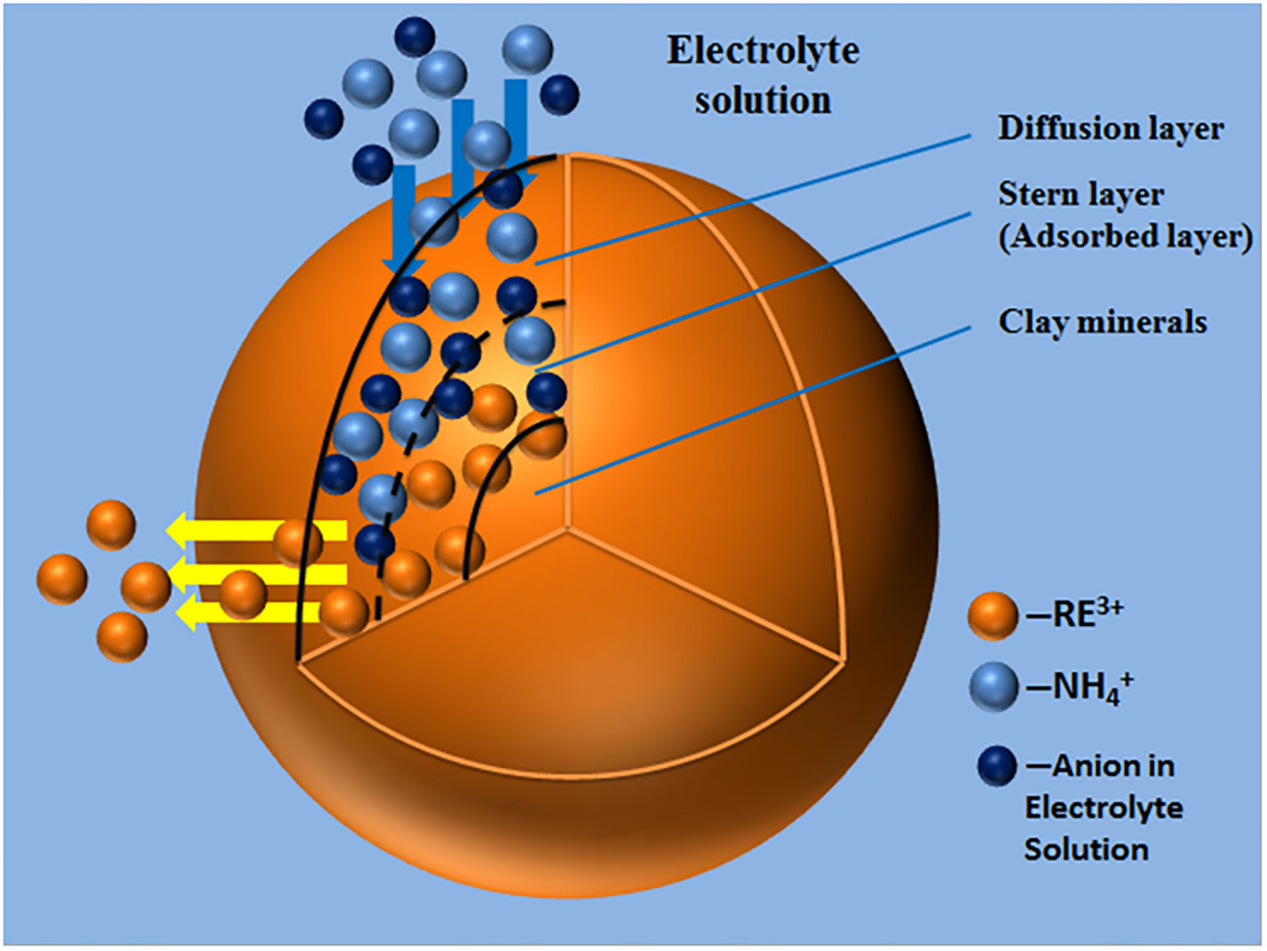
Ion exchange process.

The ion exchange process of ammonium ions in the electrolyte solution and rare earth ions, which are adsorbed on the surface of the clay minerals, is illustrated in Figure 5.

When rare earth ions are adsorbed on the surface of clay minerals in contact with the ammonium chloride solution, the ammonium hydrate ions were mainly distributed within the diffusion layer of the double electric layer. In fact, the ion exchange process of ammonium ions with rare earth ions was a thermodynamic non-spontaneous process. Increasing the concentration of ammonium chloride, high concentrations of ammonium hydrate ions have the chemical potential to make it from the diffusion layer, migrating to the stern layer, and further cross through the fixed layer to then, according to the concentration of advantage, gather near the surface of the clay minerals in the weathered crust elution-deposited rare earth ore. The large amount of ammonium ions formed a multilayer adsorption, competing for the original adsorption sites that are occupied by the hydration of rare earth ions to promote the rare earth ions desorption; the excluded rare earth ion of monolayer adsorption gradually migrated out of the fixed layer and diffusion layer and spread to the leaching agent solution. In a high concentration electrolyte solution, the zeta potentials of the clay mineral particles were not very positive because there were more hydrated ions in the tight layer. By separating the bulk solution containing rare earth ions, the leaching process of rare earth can be realized, and clay minerals loaded with ammonium ions can be obtained.

### Effects of Cation on Rare Earth Leaching in Weathered Crust Elution-Deposited Rare Earth Ore

#### Effects of Cation on Rare Earth Leaching Efficiency

**Figure 6:**
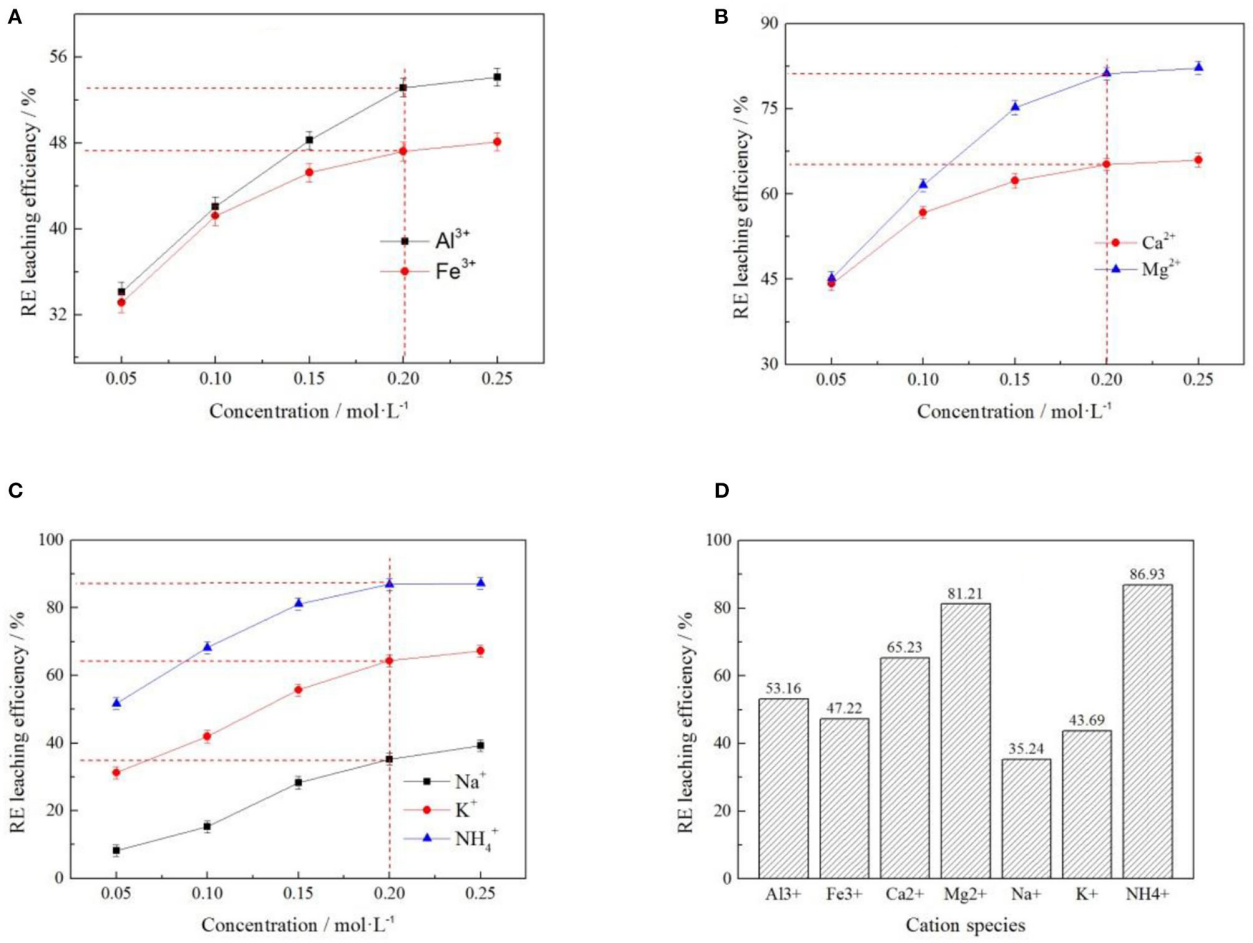
Effect of cation concentration on rare earth leaching efficiency [where **(A?C)** represent +3, +2, and +1 valence cations respectively, **(D)** represents the rare earth leaching efficiency when the concentration of various cations was 0.2 mol/L].

In order to explore the influence of different cations on rare earth leaching efficiency, *Al*^3+^, *Fe*^3+^, *Ca*^2+^, *Mg*^2+^, *Na*^+^, *K*^+^, and $NH_{4}^{+}$ 7 cations were selected to recover the rare earth from the rare earth ore samples under the same anion of *Cl*^−^. The effects of cations at different concentrations on rare earth leaching efficiencies were investigated, as shown in Figures 6A–C. At a cation concentration of 0.2 mol/L, the rare earth leaching efficiencies in all electrolyte solutions were compared, as shown in Figure 6D.

As can be seen from Figure 6, the rare earth leaching efficiency gradually increased with the increase of cation concentration. When the cation concentration exceeded 0.2 mol/L, the rare earth leaching efficiencies gradually stabilized. This is because when the electrolyte concentration reached a certain value, most of the adsorption sites on the clay mineral surface were occupied by the cation of the leaching agent. The promotion effect of rising electrolyte concentration on rare earth leaching was limited (Wang et al., 2018). Besides, it can be seen from Figures 6A–C that the electrolyte cations with the highest rare earth leaching efficiency among the three valence cations was $NH_{4}^{+}$. It can be seen from Figure 6D that, under the same electrolyte concentration, $NH_{4}^{+}$ showed a stronger leaching effect of rare earth than *Mg*^2+^ and *Al*^3+^, and the leaching efficiencies of rare earth were 86.93, 81.21, and 53.16%, respectively, indicating that $NH_{4}^{+}$ had the best leaching effect on rare earth under the same anion.

#### Effects of Cations on Zeta Potential of Clay Minerals

**Figure 7:**
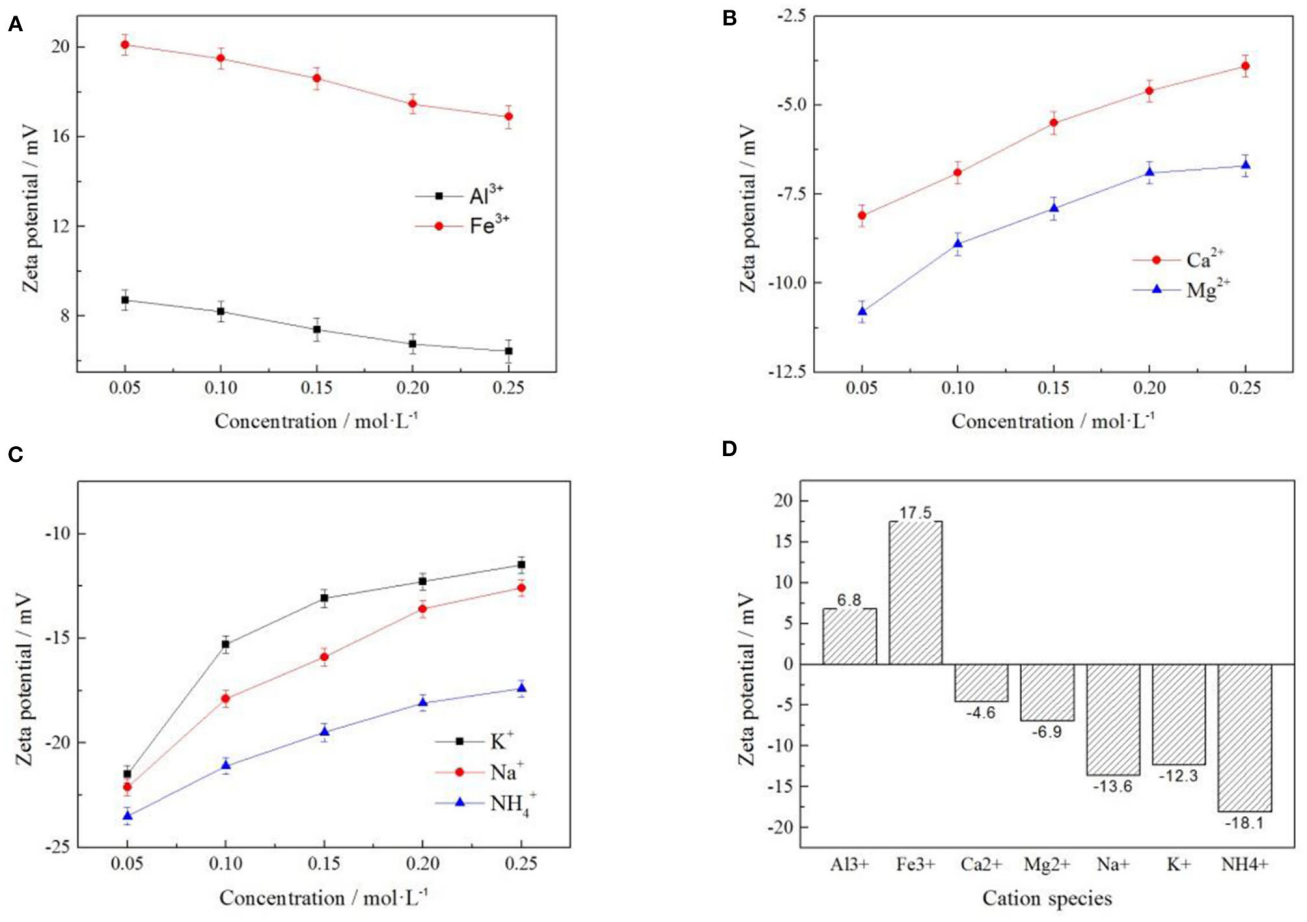
Effect of cation concentration on zeta potential of clay minerals [where **(A?C)** represent +3, +2, and +1 valence cations respectively, **(D)** represents the zeta potential of clay minerals when the concentration of various cations was 0.2 mol/L].

In order to understand the effect of cations on zeta potential of clay mineral surface, *Al*^3+^, *Fe*^3+^, *Ca*^2+^, *Mg*^2+^, *Na*^+^, *K*^+^, and $NH_{4}^{+}$ 7 cations were selected under the same anion of *Cl*^−^. The effects of cations on zeta potential on clay mineral surface at different cation concentrations were investigated, as shown in Figures 7A–C. When the cation concentrations were 0.2 mol/L, the zeta potentials of clay minerals under different electrolyte solutions were compared, as shown in Figure 7D.

As can be seen from Figure 7, the zeta potential on the clay mineral surface decreased with the increase of electrolyte concentration in +3-valent cationic electrolyte solution. In +2 and +1 valence cationic electrolyte solutions, zeta potential on the clay mineral surface increased with the increase of electrolyte solution concentration. Different cations have a greater impact on zeta potential, and cations in the same valence state have different zeta potentials, which was mainly due to the difference in the ion radius leading to different zeta potentials (Zhang Z. Y. et al., 2018). According to Figure 7D, by comparing the zeta potential of clay minerals at the same concentration of each electrolyte cation, the $NH_{4}^{+}$ absolute value of zeta potential was the largest, and the zeta potential value was negative. It was therefore easier to exchange the rare earth ions from weathered crust elution-deposited rare earth ore, verifying the ion-exchange effect of $NH_{4}^{+}$ with rare earth ions was better than other cations.

#### Correlation Between Rare Earth Leaching Efficiency and Zeta Potential Under Different Cation

**Figure 8:**
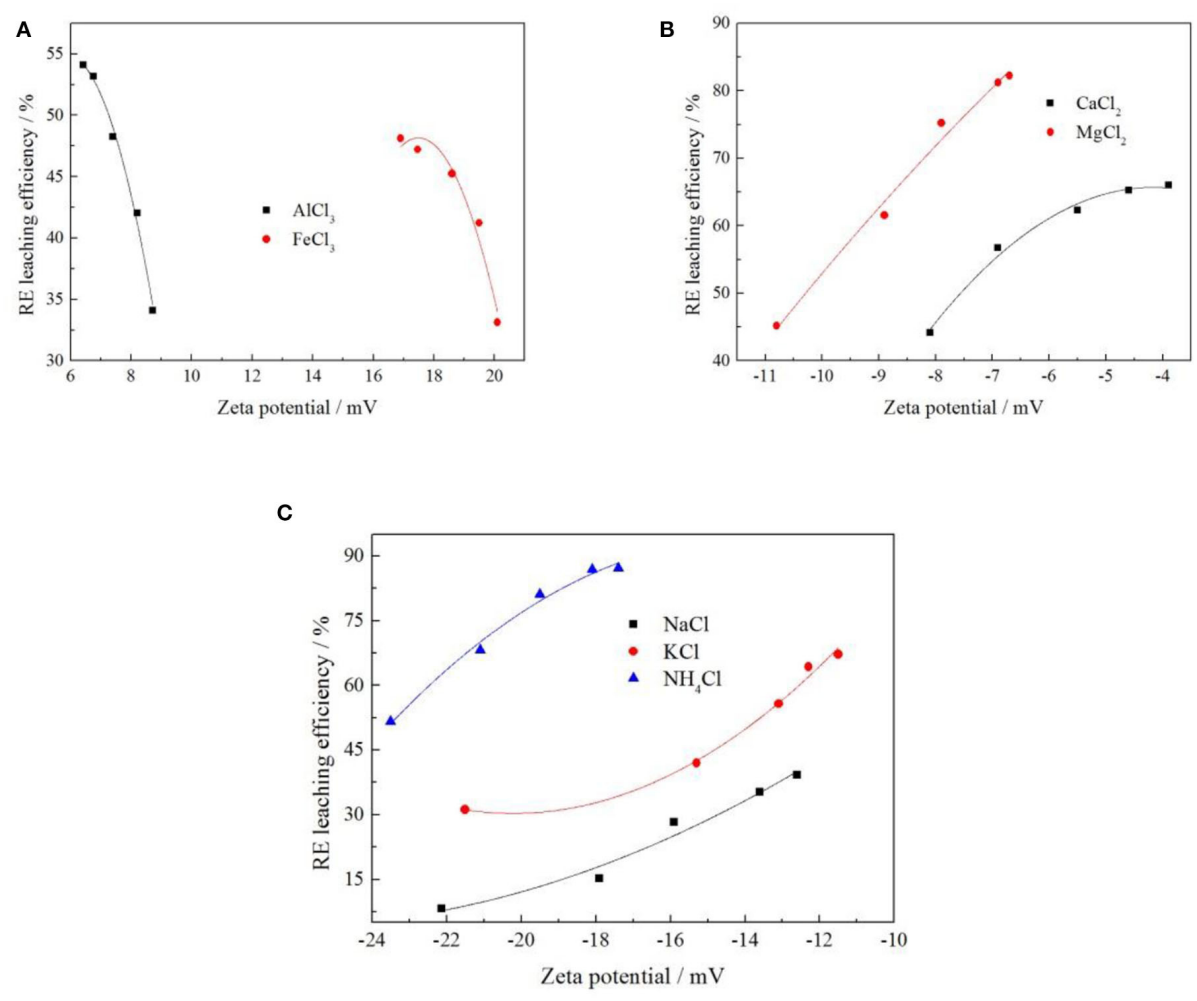
Fitting of zeta potential and leaching efficiency under different cation [where **(A–C)** represent +3, +2, and +1 valence cations, respectively].

In order to explore the relationship between the zeta potential on the clay mineral surface and the rare earth leaching efficiency, seven cations with different valence states were selected under the same anion of *Cl*^−^. The zeta potential and rare earth leaching efficiency under each electrolyte were presented through electronic double-layer model, as shown in Figure 8. The fitting equations of zeta potential with leaching efficiency was shown in Table 2.

**Table 2:** The fitting equation of cationic zeta potential—leaching efficiency.

**Cation**	**Fitting equation**	**R^2^**
*Al*^3+^	Y = −2.52X^2^ + 29.60X – 31.96	0.98903
*Fe*^3+^	Y = −2.08X^2^ + 72.77X – 588.15	0.94190
*Ca*^2+^	Y = −1.36X^2^ – 11.35X + 42.03	0.98323
*Mg*^2+^	Y = −0.27X^2^ + 4.50X + 125.45	0.97740
*Na*^+^	Y = 0.18X^2^ + 9.62X + 132.78	0.94684
*K*^+^	Y = 0.51X^2^ + 20.45X + 237.01	0.98285
$\;NH_{4}^{+}$	Y = −0.48X^2^ – 13.51X – 1.58	0.98133

When the cation concentrations were 0.05, 0.10, 0.15, 0.20, and 0.25 mol/L, respectively, various rare earth leaching efficiencies and zeta potentials under different leaching conditions were shown in the above Figure 8. It can be seen from Figure 8 that the zeta potential of clay minerals had a curve relationship with the rare earth leaching efficiency, which conforms to the Quadratic function fitting of the curve Y = a + bX + cX^2^ (Yang et al., 2018; Qiu et al., 2019); this was related to the characteristic adsorption between ions. Under the conditions of trivalent cations *Al*^3+^ and *Fe*^3+^ electrolyte solution, the leaching efficiency of rare earth decreased with the increase of zeta potential of clay mineral in the leaching process. Under +1 and +2 valence cationic electrolyte solutions, the rare earth leaching efficiency increased with the zeta potential of the clay mineral. The leaching efficiency of rare earth increased with the decrease of the absolute value of zeta potential of clay minerals in electrolyte solution. Different cation exchange capacities of rare earth were different, and this exchangeable capacity was manifested in different cation valence states: *Al*^3+^ > *Fe*^3+^, *Mg*^2+^ > *Ca*^2+^, $NH_{4}^{+}>K^{+}>Na^{+}$. By comparing the three curves corresponding to *Al*^3+^, *Mg*^2+^, and $NH_{4}^{+}$, the rare earth leaching efficiency of $NH_{4}^{+}$ was the highest, and the corresponding zeta potential was negative with the largest absolute value. $NH_{4}^{+}$ was therefore selected to have the best effect on the rare earth leaching process.

### Effects of Anion on Rare Earth Leaching in Weathered Crust Elution-Deposited Rare Earth Ore

#### Effect of Anion on Rare Earth Leaching Efficiency

**Figure 9:**
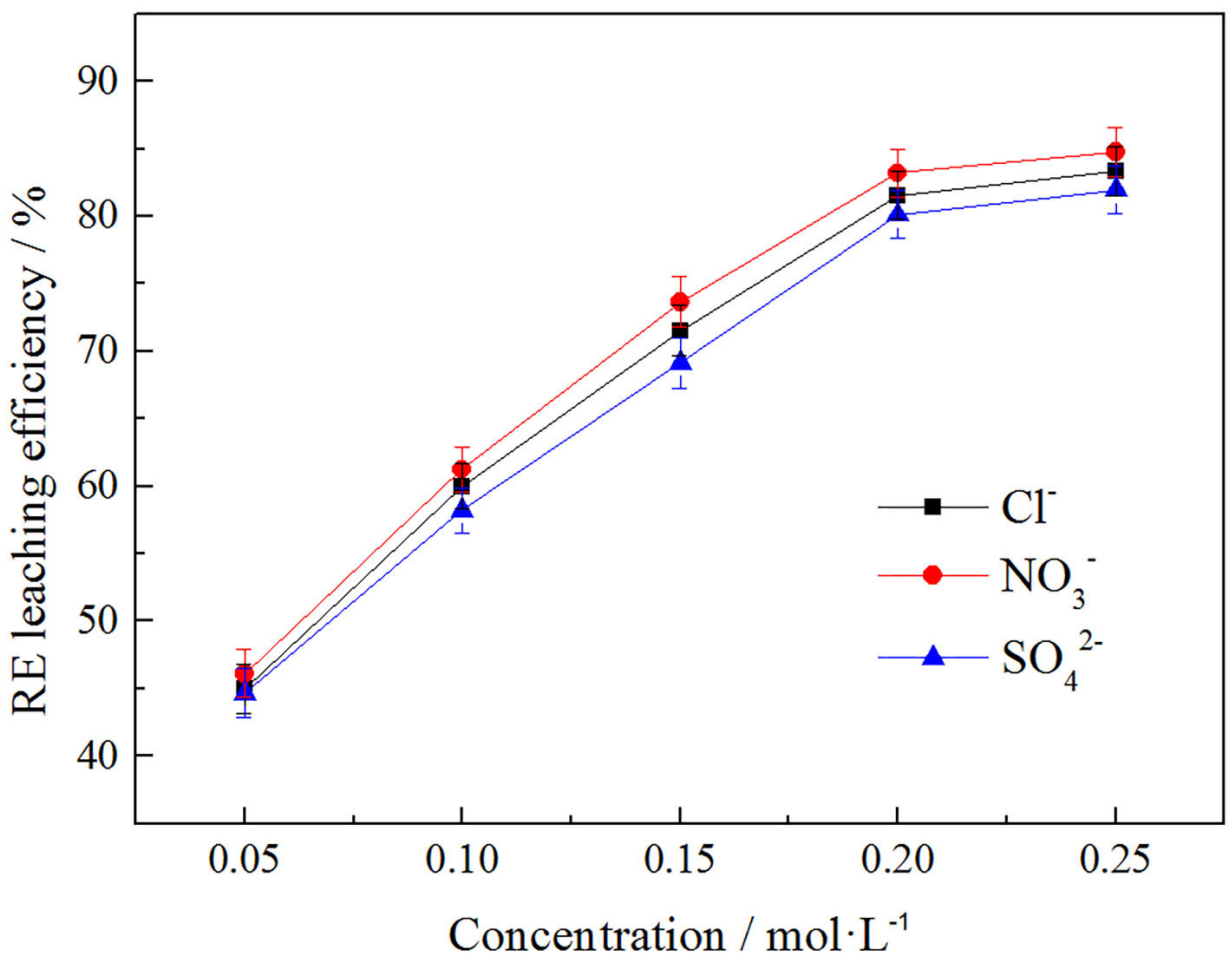
Effect of anion concentration on rare earth leaching efficiency.

In order to explore the influence of anion concentration on rare earth leaching efficiency, *Cl*^−^ $NO_{3}^{-}$, and $SO_{4}^{2-}$, three anions were selected under the same cation of $NH_{4}^{+}$ to investigate the effect of their different concentrations on rare earth leaching efficiency. The result was shown in Figure 9.

It can be seen from Figure 9 that the rare earth leaching efficiency increased with the increase of anionic concentration. When the anion concentrations were in the range of 0.05–0.2 mol/L, the rare earth leaching efficiencies increased rapidly. When the anionic concentration was >0.2 mol/L, the growth rate of rare earth leaching efficiencies was gradually stable. Therefore, 0.2 mol/L was the optimal leaching concentration. The order of influence of three kinds of anions on rare earth leaching efficiencies as follows: $NO_{3}^{-}>Cl^{-}>SO_{4}^{2-}$. The rare earth leaching efficiency were 83.21, 81.52, and 80.12%, respectively. $NO_{3}^{-}$ therefore had the best effect on the rare earth leaching process. In actual production, nitrate product belongs to restricted product, so *Cl*^−^, which had little difference in leaching efficiency of rare earth, was normally chosen in the industry.

#### Effects of Anions on Zeta Potential of Clay Minerals

**Figure 10:**
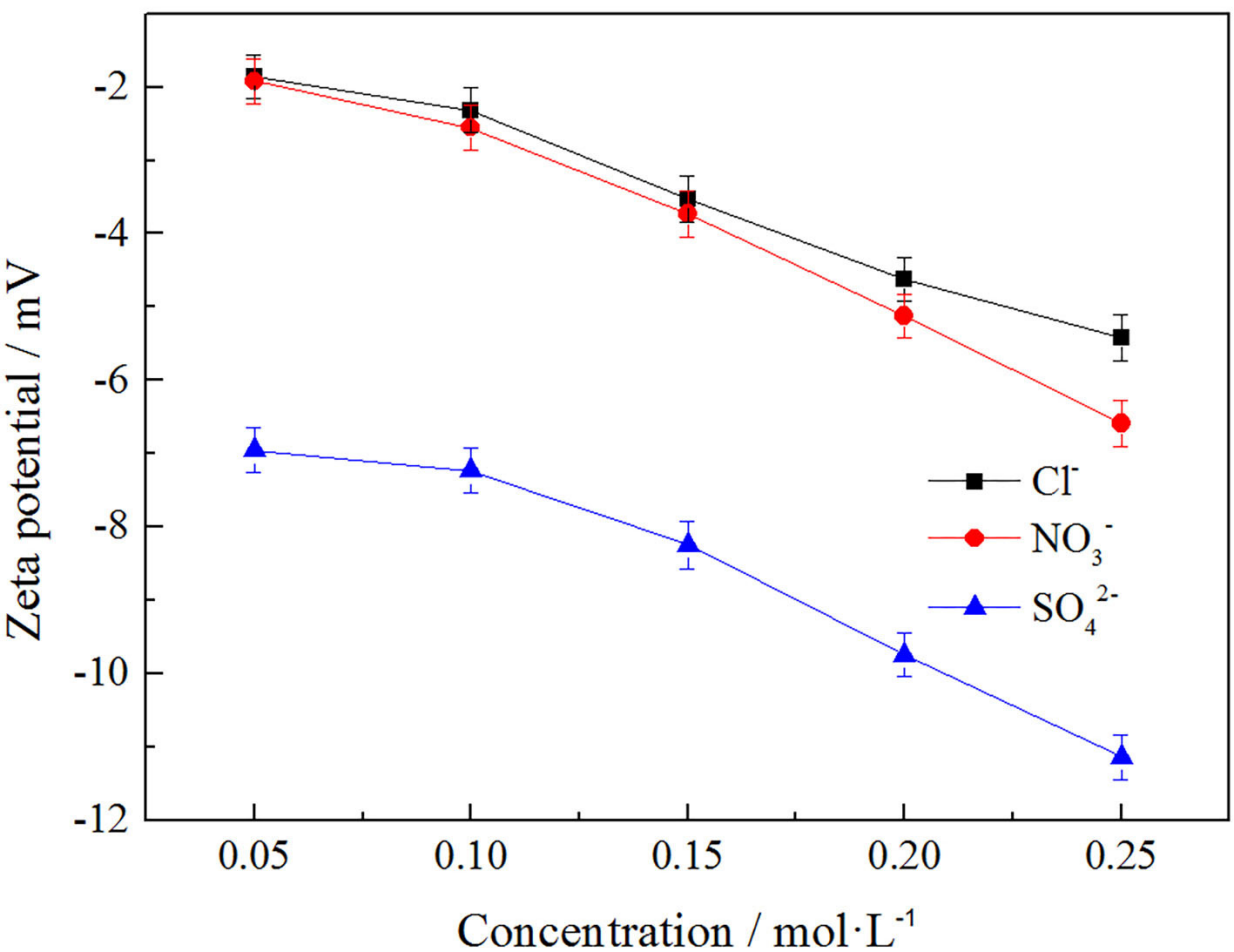
Effect of anion concentration on surface potential of clay minerals.

In order to explore the effects of anion concentration on the zeta potential of clay minerals, *Cl*^−^, $NO_{3}^{-}$, and $SO_{4}^{2-}$, three anions were selected under the same cation of $NH_{4}^{+}$ to investigate the effects of these three anions on zeta potential of clay minerals at different concentrations. The result is shown in Figure 10.

As can be seen from Figure 10, the zeta potential of the clay mineral decreased with the increase of anion concentration, while the absolute value of zeta potential of the clay mineral gradually increased. The zeta potential order of the three kinds of anions on the clay mineral was ${SO_{4}^{2-}>NO}_{3}^{-}>Cl^{-}$, and this was because $SO_{4}^{2-}$ itself contains more negative charges, which resulted in a higher zeta potential value, *Cl*^−^ and $NO_{3}^{-}$ with the same negative charges having a similar effect on zeta potential, effects of binding anions on rare earth leaching efficiency, and $NO_{3}^{-}$ having the best effect on rare earth leaching process. But in the actual production, the nitrate product mostly belongs to the controlled product, and we therefore chose *Cl*^−^, which made little difference to the leaching efficiency.

#### Correlation Between RE Leaching Efficiency and Zeta Potential Under Different Anion

**Figure 11:**
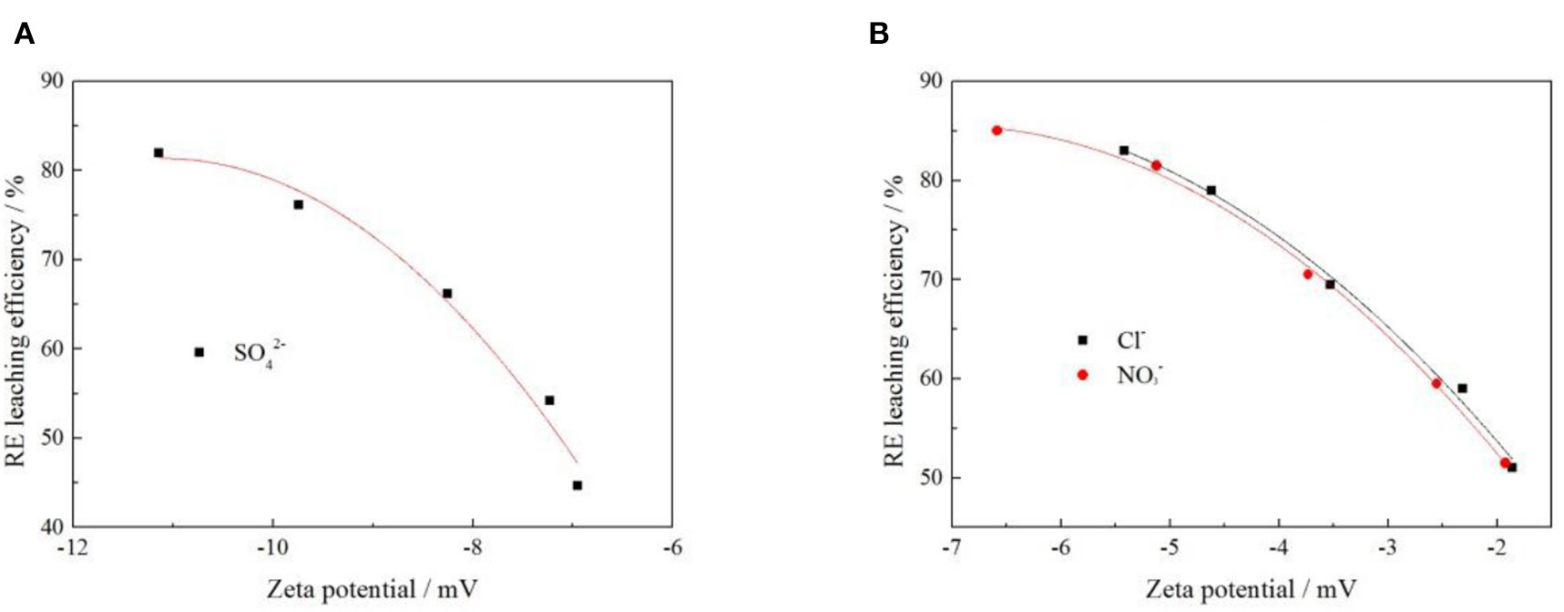
Fitting of zeta potential and leaching efficiency under different anion [where **(A,B)** represent −2 and −1 valence anions, respectively].

In order to explore the relationship between zeta potential of clay mineral and rare earth leaching efficiency under different anion. The relationship between rare earth leaching efficiency and zeta potential was fitted when the electrolyte solution anions were *Cl*^−^, $NO_{3}^{-}$, and $SO_{4}^{2-}$. The fitting results are shown in Figure 11, and the fitting equations are shown in Table 3.

**Table 3:** The fitting equation of anionic potential—leaching efficiency.

**Anion**	**Fitting equation**	**R^2^**
*Cl*^−^	Y = −1.22 X^2^ – 17.64X + 23.29	0.99010
$NO_{3}^{-}$	Y = −1.31 X^2^ – 18.33X + 20.99	0.99675
$SO_{4}^{2-}$	Y = −1.99 X^2^ – 44.23X – 163.93	0.96463

It can be seen from Figure 11 that under each electrolyte solution, the zeta potential of clay minerals had a curve relationship with the rare earth leaching efficiency, which conformed to the quadratic function fitting of the curve Y = aX^2^ + bX + c, and the fitting equations are shown in Table 3. Figure 11 shows that the rare earth leaching efficiency decreased with the increase of zeta potential of clay minerals. The greater the absolute value of zeta potential of the clay mineral with the higher the rare earth leaching efficiencies. Because there were more exchangeable ions on the surface of the clay minerals, more rare earth ions were exchanged by the electrolyte solution. In Figure 11B, there was no significant difference between *Cl*^−^ and $NO_{3}^{-}$ on zeta potential and rare earth leaching efficiency of clay minerals. In the actual production process, the nitrate product belongs to controlled products. When there were only negative monovalent anions, *Cl*^−^ was selected as the electrolyte anion to achieve better leaching effect of rare earth. When the $SO_{4}^{2-}$ acted as an anion in the electrolyte solution, the zeta potential of clay minerals was relatively high due to its large negative charge. However, from the perspective of the influence of anions on the leaching efficiency of rare earth, the rare earth leaching efficiency of *Cl*^−^ as an electrolyte anion was higher than $SO_{4}^{2-}$; when *Cl*^−^ was available as an electrolyte anion, *Cl*^−^ can thus be preferred.

### Effect of Different Ions on Swelling of Weathered Crust Elution-Deposited Rare Earth Ores

#### Effect of Different Cation on Swelling Ratio of Clay Minerals

**Figure 12:**
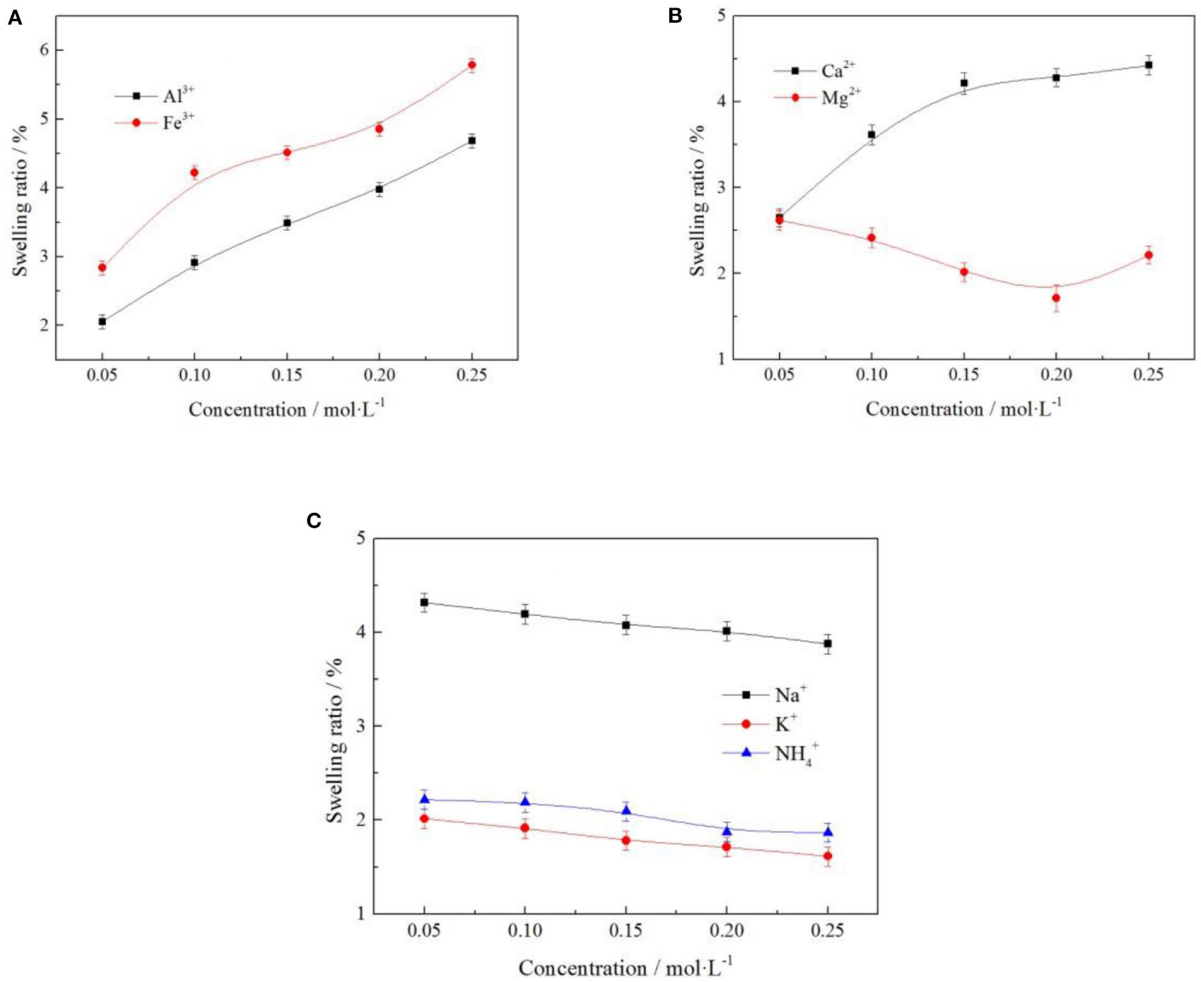
Effect of cation on swelling ratio of clay minerals [where **(A–C)** represent +3, +2, and +1 valence cations, respectively].

To understand the effects of the different cation and cation concentration on the swelling ratio of the clay minerals, the samples were subjected to various cation and cation concentrations with the same anion. Swelling ratios were constructed using the experimental data, and the results are shown in Figure 12.

It can be seen from Figure 12 when the cation of electrolyte solution for *Al*^3+^, *Fe*^3+^, and *Ca*^2+^ clay minerals swelling ratio increased as the concentration of electrolyte solution increased, as when the cation concentration increases, more cations were exchanged into the layered structure of clay mineral content. Due to ionic radius of *Al*^3+^, *Fe*^3+^, and *Ca*^2+^ were bigger, leading to the rise in swelling ratio of the clay minerals. When the cation of the electrolyte solution for *Mg*^2+^, *Na*^+^, *K*^+^, and $NH_{4}^{+}$ clay mineral swelling ratio decreased as the concentration of electrolyte solution increased, the ion displayed a certain inhibition in terms of the swelling of clay minerals. Under the optimal electrolyte concentration of 0.2 mol/L, the swelling ratio of $NH_{4}^{+}$ and *K*^+^ to clay minerals were 1.874 and 1.714%, respectively. In the actual production, $NH_{4}^{+}$ was easy to obtain and had low cost, so $NH_{4}^{+}$ was selected as the electrolyte cation with better technological value.

#### Effect of Different Anions on Swelling Ratio of Clay Minerals

**Figure 13:**
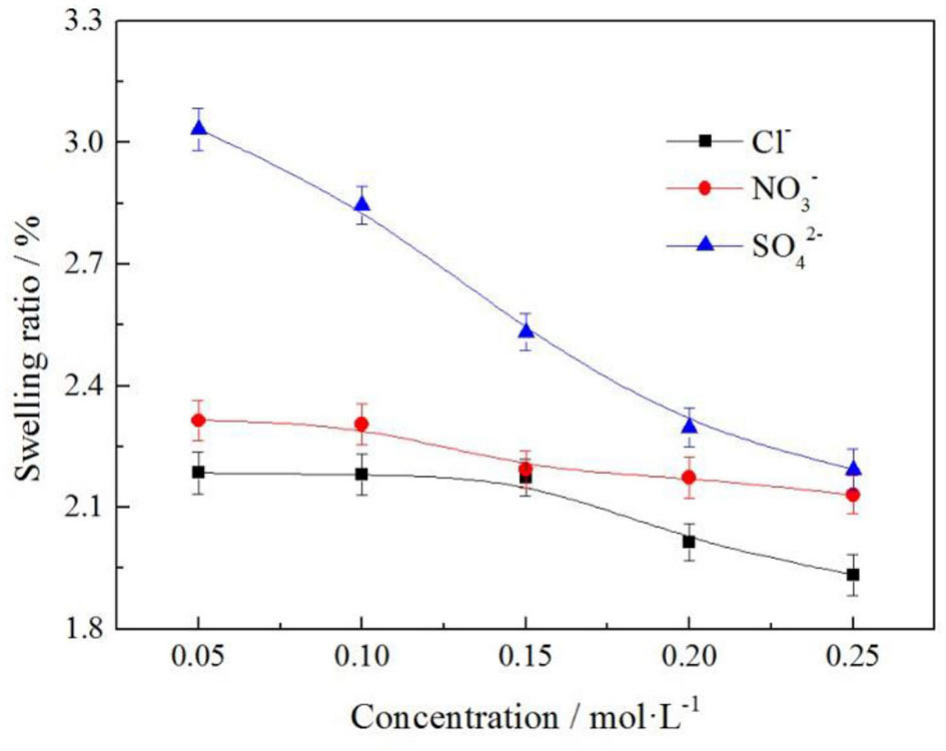
Effect of anion on swelling ratio of clay minerals.

To understand the effects of the different anion and anion concentration on the swelling ratio of the clay minerals, the samples were subjected to various anion and anion concentration with the same cation. Swelling ratios were constructed using the experimental data, and the results are shown in Figure 13.

It can be seen from Figure 13 that the swelling ratio of clay minerals decreases with the increase of electrolyte solution anion concentration. The order of swelling ratio of the three types of anions to clay minerals was as follows: $SO_{4}^{2-}>NO_{3}^{-}>Cl^{-}$. When the electrolyte solution concentration was 0.2 mol/L, the swelling ratios of clay minerals were 2.297, 2.174, and 2.015%, respectively; when the anion was *Cl*^−^, the swelling ratio of clay minerals was 2.015% at the minimum, and the rare earth leaching efficiency was 81.52% in this case. This indicates that in actual production, the anion selection *Cl*^−^ of electrolyte solution had the least influence on mine swelling but had a high rare earth leaching efficiency.

## Conclusions

In the study of the influence of anion and cation on weathered crust elution-deposited rare earth ore, it was found that the order of the rare earth leaching ability in three valence cations of $NH_{4}^{+}>K^{+}>Na^{+}$, *Mg*^2+^ > *Ca*^2+^, *Al*^3+^ > *Fe*^3+^ and the $NH_{4}^{+}$ were the most affected electrolyte cations on rare earth leaching efficiency; rare earth leaching efficiency was 86.93% at the optimal leaching concentration. The influence of the three anions on the leaching efficiency of rare earth was as follows: $NO_{3}^{-}>Cl^{-}>SO_{4}^{2-}$. The leaching efficiencies of rare earth were 83.21, 81.52, and 80.12% at the optimal leaching concentration. Meanwhile, in the study of zeta potential on clay mineral surface, it was found that $\;NH_{4}^{+}$ had the greatest effect on zeta potential in the influence of different cations on zeta potential of weathered crust elution-deposited rare earth ore, and the zeta potential was −18.1 mV at the optimal leaching concentration. The order of the effect of three anions on zeta potential was ${SO_{4}^{2-}>NO}_{3}^{-}>Cl^{-}$ in terms of the influence of anions on zeta potential of a weathered crust elution-deposited rare earth ore. Considering the relation between rare earth leaching efficiency and zeta potential, $NH_{4}^{+}$ was selected as the electrolyte cation to have the best effect on rare earth leaching process, while the anion was selected as $NO_{3}^{-}$ to have the best effect. But in the actual production, the nitric acid product mostly belongs to the controlled product and therefore chose the *Cl*^−^, which made little difference to the leaching efficiency. To sum up, when considering which electrolyte solution to use for cation and anion alone, the cation chooses $NH_{4}^{+}$, the anion chooses *Cl*^−^, and the relationship between the rare earth leaching efficiency and zeta potential conforms to the following equations: $NH_{4}^{+}$:Y = −0.48X^2^ – 13.51X – 1.58, *R*^2^ = 0.98133; *Cl*^−^:Y= −1.22 X^2^ – 17.64X + 23.29, and *R*^2^ = 0.99010. In the experiment of the effect of various ions on the swelling of weathered crust elution-deposited rare earth ore, it is found that $NH_{4}^{+}$ and *Cl*^−^ have the least influence on the swelling of clay minerals, and the swelling ratios of $NH_{4}^{+}$ and *Cl*^−^ under the optimal rare earth leaching concentration were 1.874 and 2.015%, respectively.

## Data Availability Statement

The original contributions presented in the study are included in the article/supplementary materials, further inquiries can be directed to the corresponding author.

## Author Contributions

ZZ and RC designed the project. WC performed the experiments. ZZ and WC analyzed the data. WC, ZZ, and ZC wrote the manuscript. All authors contributed to the article and agreed to the published version of the manuscript.

## Conflict of Interest

The authors declare that the research was conducted in the absence of any commercial or financial relationships that could be construed as a potential conflict of interest.
